# Efficacy of EGFR‐TKIs with or without upfront brain radiotherapy for *EGFR*‐mutant NSCLC patients with central nervous system metastases

**DOI:** 10.1111/1759-7714.13189

**Published:** 2019-09-10

**Authors:** Yu Saida, Satoshi Watanabe, Tetsuya Abe, Satoshi Shoji, Koichiro Nozaki, Kosuke Ichikawa, Rie Kondo, Kenichi Koyama, Satoru Miura, Hiroshi Tanaka, Masaaki Okajima, Masaki Terada, Takashi Ishida, Hiroki Tsukada, Masato Makino, Akira Iwashima, Kazuhiro Sato, Naoya Matsumoto, Hirohisa Yoshizawa, Toshiaki Kikuchi

**Affiliations:** ^1^ Department of Respiratory Medicine and Infectious Diseases Niigata University Graduate School of Medical and Dental Sciences Niigata Japan; ^2^ Department of Respiratory Medicine, Shinrakuen Hospital Niigata Japan; ^3^ Department of Internal Medicine, Niigata Cancer Center Hospital Niigata Japan; ^4^ Department of Respiratory Medicine, Saiseikai Niigata Hospital Niigata Japan; ^5^ Department of Respiratory Medicine, Niigata Prefectural Central Hospital Joetsu Japan; ^6^ Department of Respiratory Medicine, Niigata City General Hospital Niigata Japan; ^7^ Department of Respiratory Medicine, Shibata Hospital Shibata Japan; ^8^ Department of Respiratory Medicine, Nagaoka Chuo General Hospital Nagaoka Japan; ^9^ Department of Respiratory Medicine, Nagaoka Red Cross Hospital Nagaoka Japan; ^10^ Department of Respiratory Medicine, Nishi‐Niigata Chuo National Hospital Niigata Japan; ^11^ Department of Respiratory Medicine, Niigata Medical Center Niigata Japan

**Keywords:** Brain metastases, *EGFR* mutation, EGFR tyrosine kinase inhibitor, non‐small cell lung cancer, radiotherapy

## Abstract

**Background:**

Although the clinical efficacy of epidermal growth factor receptor tyrosine kinase inhibitors (EGFR‐TKIs) in *EGFR*‐mutant non‐small cell lung cancer (NSCLC) patients has been demonstrated, their efficacy in *EGFR*‐mutant NSCLCs with central nervous system (CNS) metastases and the role of radiotherapy remain unclear. This study aimed to determine if it is preferable to add upfront cranial radiotherapy to EGFR‐TKIs in patients with *EGFR*‐mutant NSCLC with newly diagnosed brain metastases.

**Methods:**

We retrospectively analyzed the data of *EGFR*‐mutant NSCLC patients with CNS metastases who received EGFR‐TKIs as a first‐line therapy.

**Results:**

A total of 104 patients were enrolled and 39 patients received upfront brain radiotherapy, while 65 patients received first and second generation EGFR‐TKIs first. The median time to treatment failure (TTF) was 7.8 months (95% confidence interval [CI]: 6.3–9.4). The median survival time (MST) was 24.0 months (95% CI: 20.1–30.1). The overall response rate of the CNS was 37%. The median CNS progression‐free survival (PFS) was 13.2 months (95% CI: 10.0–16.2). Brain radiotherapy prior to EGFR‐TKI prolonged TTF (11.2 vs. 6.8 months, *P* = 0.038) and tended to prolong CNS‐PFS (15.6 vs. 11.1 months, *P* = 0.096) but was not significantly associated with overall survival (MST 26.1 vs. 24.0 months, *P* = 0.525). Univariate and multivariate analyses indicated that poor performance status and the presence of extracranial metastases were poor prognostic factors related to overall survival.

**Conclusion:**

EGFR‐TKI showed a favorable effect for *EGFR*‐mutant NSCLC patients with CNS metastases. Prolonged TTF and CNS‐PFS were observed with upfront brain radiotherapy.

## Key points

### Significant findings of the study

In this retrospective study of *EGFR*‐mutant NSCLC patients with brain metastases who received EGFR‐TKIs for a first‐line drug therapy, upfront brain radiotherapy and TKI showed prolonged time to treatment failure compared to TKI alone.

### What this study adds

This finding might support clinician's choice to give upfront brain RT for *EGFR*‐mutated NSCLC patients with brain metastases.

## Introduction

The central nervous system (CNS) is a common site of metastasis of non‐small cell lung cancer (NSCLC). Brain metastases (BMs) are observed in approximately 10% of NSCLC patients at diagnosis and occur in 25%–30% of these patients throughout their clinical courses.^1^, ^2^ The complication of CNS metastasis is a poor prognostic factor in NSCLC patients. The prognosis for advanced NSCLC patients with CNS metastases is approximately 4–11 weeks if untreated.^1^, ^2^, ^3^

A retrospective analysis showed that patients with epidermal growth factor receptor (*EGFR)* ‐mutant NSCLC were more likely to develop BMs than those with *EGFR*‐wild‐type NSCLC (31.4% vs. 19.7%, *P* < 0.001); moreover, BMs from *EGFR*‐mutant NSCLC often disseminate (30.8% vs. 12.7%).^4^ Ultimately, CNS metastases can become lethal in *EGFR*‐mutant NSCLC patients. However, according to a previous study, survival after BMs was significantly longer for *EGFR*‐mutant cases than for *EGFR*‐wild‐type cases (hazard ratio: 2.23; 95% CI: 1.62–3.10, *P* < 0.001). This finding has been attributed to the benefit of EGFR‐tyrosine kinase inhibitors (TKIs) for *EGFR*‐mutant patients. EGFR‐TKIs such as gefitinib, erlotinib, and afatinib have been established as standard first‐line drug therapies in advanced NSCLC patients harboring *EGFR* mutations on the basis of a number of phase III trials.^5^, ^6^, ^7^, ^8^, ^9^, ^10^, ^11^

There is no consensus regarding the management of *EGFR*‐mutant NSCLC patients with BMs. The concentration of first‐generation EGFR‐TKIs in cerebrospinal fluid was much lower than that in plasma.^12^ Although several prospective trials examined the effect of EGFR‐TKIs on BMs without brain radiotherapy (RT), the results from these studies were inconclusive.^13^, ^14^, ^15^ Traditionally, patients with multiple BMs have been treated with whole‐brain radiotherapy (WBRT). Previous reports examining the efficacy of EGFR‐TKIs plus WBRT have provided conflicting results.^16^, ^17^ Therefore, whether WBRT in addition to EGFR‐TKI is especially needed for patients with asymptomatic BMs is unclear. Recently, evidence regarding the efficacy of stereotactic radiosurgery (SRS) for a few (four or less) small (< 3 cm) brain metastases has been gradually accumulated.^18^, ^19^ A retrospective study of *EGFR*‐mutant NSCLC patients with BMs suggested increased survival with a combination of upfront SRS and EGFR‐TKI compared to that with TKI monotherapy.^20^

The aim of this retrospective study was to determine the impact of adding upfront cranial radiotherapy to EGFR‐TKI in patients with *EGFR*‐mutant NSCLC with newly diagnosed brain metastases on time to treatment failure, intracranial progression‐free survival, and overall survival. We also evaluated the prognostic factors that could affect the treatment outcome.

## Methods

### Study design and patients

We retrospectively evaluated patient clinical outcomes and background for seven years between January 2008 and December 2014 in Niigata Lung Cancer Treatment Group facilities. The eligibility criteria were as follows: (i) pathologically confirmed NSCLC at stage IV, postoperative recurrence, or recurrence after treatment for locally advanced cancer; (ii) newly radiographically diagnosed brain metastasis and/or leptomeningeal metastases; (iii) confirmed *EGFR* mutation at any point during the clinical course; and (iv) initiated EGFR‐TKI as a first‐line drug therapy during the above period.

The study protocol was approved by the institutional review board of each participating institution. The requirement for patient consent was waived due to the retrospective nature of the study and lack of potential harm to patients. The following variables were collected for analysis: age, sex, Eastern Cooperative Oncology Group‐Performance Status (ECOG‐PS), Karnofsky Performance Status (KPS), smoking history, pathological diagnosis, stage, *EGFR* mutation status, initial CNS symptom, number of BMs, size of the largest BM, presence of leptomeningeal metastases, extracranial metastases, kind of EGFR‐TKI, type of brain RT, site of disease progression, cause for discontinuation of EGFR‐TKI, 2nd line therapy, and cause of death. The date of initial EGFT‐TKI treatment, RT, discontinuation of systemic disease progression, CNS disease progression, most recent follow‐up and death were also recorded.

### Treatment and response evaluation

Patients in this study were treated with EGFR‐TKI including gefitinib 250 mg, erlotinib 150 mg, or afatinib 40 mg once a day. WBRT was usually performed at a dose of 30 Gy in 10 fractions. For stereotactic irradiation (STI) of the brain, SRS or stereotactic radiotherapy (SRT) was performed. With SRS, the full radiation dose was delivered in one session, while with SRT, the dose of radiation was delivered over a course of several treatment sessions, instead of all at once. The median marginal dose of SRS was 20 Gy. SRT was usually performed at a dose of 30 Gy in four fractions.

At the baseline of disease diagnosis, computed tomography (CT) and/or positron emission tomography‐CT of the chest, abdomen, and pelvis, bone scintigraphy and brain magnetic resonance imaging (MRI) or brain CT were performed. CT for extracranial disease and MRI or CT for intracranial disease were usually repeated every 2–3 months. The tumor response was assessed as complete response (CR), partial response (PR), stable disease (SD), or progressive disease (PD) according to response evaluation criteria in solid tumors (RECIST) 1.1.

### Statistical analysis

The primary end point was time to treatment failure (TTF), and the secondary end points were PFS, CNS‐PFS, and overall survival (OS). TTF was defined from the initiation of treatment (EGFR‐TKI or local therapy for BM, whichever came first) to the end of treatment (disease progression, discontinuation of EGFR‐TKI from any cause, or death from any cause, whichever came first). PFS was measured from the initiation of treatment (EGFR‐TKI or local therapy for BM, whichever came first) to disease progression or death from any cause. CNS‐PFS was measured from the initiation of treatment (EGFR‐TKI or local therapy for BM, whichever came first) to CNS progression or death from any cause. OS was measured from the initiation of treatment (EGFR‐TKI or local therapy for BM, whichever came first) to death from any cause or last survival follow‐up.

We used Fisher's exact test, chi‐square test, or *t*‐test as appropriate to compare the characteristics of patients with and without upfront brain RT. Kaplan‐Meier survival curves were constructed for TTF, PFS, CNS‐PFS and OS; the differences between groups were identified using the log‐rank test. Univariate and multivariate Cox proportional hazards models were used to assess prognostic factors for OS. Each analysis was two‐sided, with a 5% significance level and 95% confidence interval (CI). All analyses were performed using JMP for Windows software version 13.0 (SAS Institute, Cary, NC).

## Results

### Patient characteristics

A total of 104 NSCLC patients harboring *EGFR* mutations with CNS metastases who underwent EGFR‐TKI for the first‐line drug therapy from 10 institutions were identified. The median follow‐up was 21.5 months (range: 0.2–83.0). The baseline and initial treatment characteristics of these patients are summarized in Table 1. All patients were from Japan. Most were female (64%), never smokers (59%), and had adenocarcinoma (98%). The two major types of mutations, exon 19 deletion and exon 21 L858R point mutation, accounted for 95% of patients. More symptomatic BMs were observed in patients who received upfront brain RT (1st RT group) than in those who did not receive upfront brain RT (1st TKI group) (51% vs. 15%, *P* = 0.0001). Additionally, patients in the 1st RT group were more likely to have larger BMs (22 mm vs. 9 mm, *P* < 0.001). There was no significant difference in ECOG‐PS, number of BMs, extracranial metastases, leptomeningeal metastases, diagnosis‐specific Graded Prognostic Assessment (DS‐GPA), or *EGFR* mutations between the groups.

**Table 1:** Patient characteristics

	Total		1st TKI		1st RT		
Characteristics	*N* = 104	%	*N* = 65	%	*N* = 39	%	*P*‐value
Sex							1.000
Male	37	36%	24	37%	14	36%	
Female	67	64%	41	63%	25	64%	
Age, median (range, years)	67	(36–91)	67	(36–86)	71	(41–91)	0.3866
Body height, median (range, cm) Bodyweight median (range, kg)	157 51	(130–180) (33–87)	156 49	(130–180) (34–87)	158 52	(130–170) (33–69)	0.7262 0.9162
Smoking history							0.8374
Never smoker	61	59%	39	60%	22	56%	
ECOG performance status							0.8468
0–1	66	63%	43	66%	23	59%	
2	20	19%	11	17%	9	23%	
3	16	15%	10	15%	6	15%	
4	2	2%	1	2%	1	3%	
Stage							0.0003
Stage IV	82	79%	59	91%	23	59%	
Recurrence after operation or (chemo) radiotherapy	22	21%	6	9%	16	41%	
Pathology							0.1383
Adeno	102	98%	65	100%	37	95%	
Adenosquamous	2	2%	0	0%	2	5%	
Number of BMs							0.8683
1	32	31%	21	32%	12	31%	
2	17	16%	9	14%	8	21%	
3	6	6%	4	6%	2	5%	
4	8	8%	6	9%	2	5%	
5≤	41	39%	25	38%	15	38%	
Size of the largest BM, median (range, mm)	12	(3–57)	9	(3–55)	22	(6–57)	<0.001
Initial brain symptom							0.0001
Symptomatic	30	29%	10	15%	20	51%	
Asymptomatic	74	71%	55	85%	19	49%	
Primary leptomeningeal carcinomatosis							1.0000
Yes	8	8%	5	8%	3	8%	
No	96	92%	60	92%	36	92%	
Extracranial metastasis							0.2481
Yes	77	74%	51	78%	26	67%	
No	27	26%	14	22%	13	33%	
DS‐GPA							0.5859
0	11	11%	6	9%	5	13%	
0.5	17	16%	12	18%	5	13%	
1	21	20%	15	23%	6	15%	
1.5	18	17%	12	18%	6	15%	
2	17	16%	7	11%	10	26%	
2.5	8	8%	6	9%	2	5%	
3	10	10%	6	9%	4	10%	
3.5	2	2%	1	2%	1	3%	
EGFR mutation							0.1383
Ex 19 deletion	52	50%	37	57%	15	38%	
Ex 21 L858R	47	45%	24	37%	23	59%	
Ex 18 L861Q	3	3%	2	3%	1	3%	
Ex 18 G719X	2	2%	2	3%	0	0%	
EGFR TKI							0.1774
Gefitinib	77	74%	53	82%	26	77%	
Erlotinib	24	23%	10	15%	12	31%	
Afatinib	3	3%	2	3%	1	3%	
Upfront brain radiotherapy							—
No	65	63%	65	100%	19	49%	
STI only	19	18%			16	41%	
WBRT only	16	15%			4	10%	
STI + WBRT	4	4%					

BM, brain metastasis; DS‐GPA, diagnosis‐specific graded prognostic assessment; ECOG, Eastern Cooperative Oncology Group; RT, radiotherapy; STI, stereotactic irradiation; TKI, tyrosine kinase inhibitor; WBRT, whole brain radiotherapy.

A total of 39 of 104 patients received upfront brain RT, while 65 patients received EGFR‐TKI therapy first. Of these 39 patients, 19 received STI (SRS and SRT), 16 received WBRT, and four received both STI and WBRT. Three of these 39 patients received EGFR TKI concurrently with WBRT, while the others received TKI therapy sequentially. Median interval between initiation of radiotherapy and initiation of EGFR TKI was 19 days (range: 0–277).

### Treatment outcome

**Figure 1:**
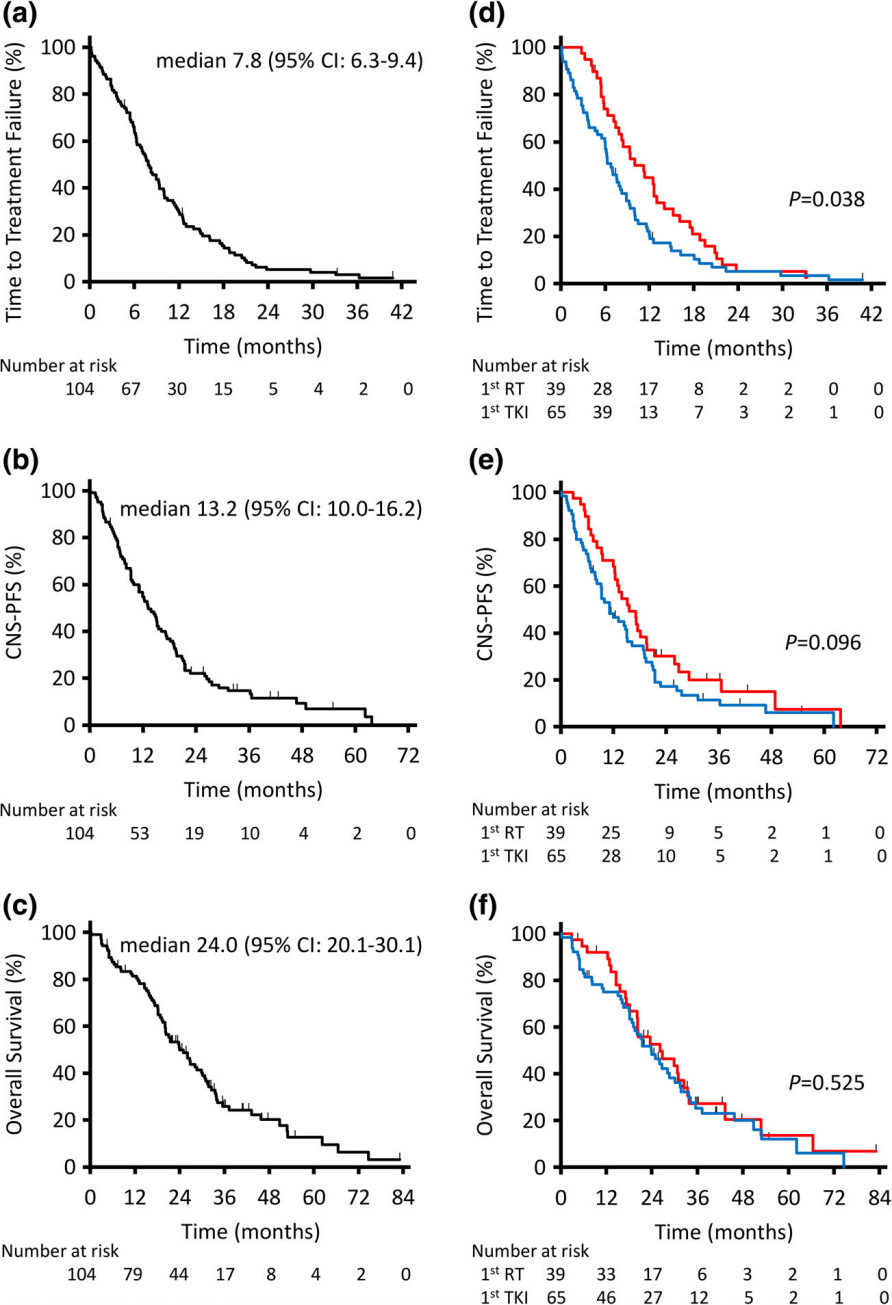
Kaplan‐Meier analysis of time to treatment failure, CNS‐PFS, and overall survival in the entire population (**a**, **b**, and **c**). Kaplan‐Meier analysis of time to treatment failure, CNS‐PFS, and overall survival compared between patients treated with upfront brain radiotherapy (RT) and those treated without upfront RT (**d**, **e**, and **f**). CI, confidence interval; CNS, central nervous system; PFS, progression‐free survival. 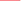 1st RT and 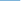 1st TKI.

The Kaplan‐Meier curve for the TTF, CNS‐PFS, and OS of the entire cohort is shown in Fig 1. The median TTF, CNS‐PFS, and OS of the entire cohort were 7.8 months (95% CI: 6.3–9.4), 13.2 months (95% CI: 10.0–16.2), and 24.0 months (95% CI: 20.1–30.1), respectively (Fig 1a–c). As shown in Fig 1d, TTF was significantly longer for patients who received brain RT prior to EGFR‐TKI (1st RT) than for those who did not receive upfront brain RT (1st TKI) (11.2 vs. 6.8 months, *P* = 0.038) (Fig 1d). For patients age <70, female, smoker, ECOG‐PS < 2, KPS > 70, max size of BMs ≥15 mm, and L858R, TTF was longer in the 1st RT group than in the 1st TKI group (Fig S1). Although nonsignificant, patients in the 1st RT group tended to have longer CNS‐PFS (15.6 vs. 11.1 months, *P* = 0.096) (Fig 1e). There was no significant difference in OS (MST 26.1 vs. 24.0 months, *P* = 0.525) (Fig 1f). Moreover, there was no significant difference in TTF, CNS‐PFS, and OS between patients who received STI and those who received WBRT as brain RT prior to EGFR‐TKI (Fig S2).

The overall response rate (ORR) and CNS‐RR of the entire cohort were 62% and 37%, respectively. There was no significant difference in ORR and CNS‐RR between the 1st RT group and the 1st TKI group (ORR; 64% vs. 60%, *P* = 0.8353, CNS‐RR; 36% vs. 37%, *P* = 1.0000) (Table 2).

**Table 2:** Treatment response

	Total	1st TKI	1st RT	
Response	*N* = 104	*N* = 65	*N* = 39	*P*‐value
Best overall response				
CR	1	0	1	—
PR	63	39	24	—
SD (include non‐CR/non‐PD)	22	13	9	—
PD	10	8	2	—
Not evaluated	8	5	3	—
Response rate	64 (62%)	39 (60%)	25 (64%)	0.8353
Disease control rate	86 (83%)	52 (80%)	34 (87%)	0.4289
Best CNS response				
CR	15	10	5	—
PR	23	14	9	—
SD (include non‐CR/non‐PD)	46	27	19	—
PD	7	6	1	—
Not evaluated	13	8	5	—
Response rate	38 (37%)	24 (37%)	14 (36%)	1.0000
Disease control rate	84 (81%)	51 (78%)	33 (85%)	0.6082

CR, complete response; PD, progression of disease; PR, partial response; RT, radiotherapy; SD, stable disease; TKI, tyrosine kinase inhibitor; WBRT, whole brain radiotherapy.

### Patterns of treatment failure

Of the 104 patients, 88 developed disease progression during their follow‐up period. Compared to the 1st RT population, the 1st TKI population was more likely to present with an intracranial first site of progression, although the difference was nonsignificant (46% vs. 28%, *P* = 0.1151) (Table 3). Among the patients who developed disease progression only in intracranial lesions, EGFR‐TKI treatment were continued beyond PD in four out of 20 in 1st TKI patients and one out of eight 1st RT patients, respectively.

**Table 3:** Patterns of disease progression

	Total		1st TKI		1st RT		
Pattern	*N* = 88	%	*N* = 56	%	*N* = 32	%	*P*‐value
Intracranial lesions only	28	32%	20	36%	8	25%	0.3482
Extracranial lesions only	52	59%	30	54%	22	69%	0.3696
Both intra‐ and extracranial lesions	7	8%	6	11%	1	3%	0.4146
Not available	1	1%	0	0%	1	3%	—
Intracranial lesion only + both lesions	35	40%	26	46%	9	28%	0.1151

TKI, tyrosine kinase inhibitor; RT, radiotherapy.

### Cause of death

Of the 104 patients, 79 died during their follow‐up period. Thirty‐one patients (39%) died of disease progression, including intracranial lesions. The cause of death was not significantly different between the 1st TKI group and the 1st RT group (Table 4).

**Table 4:** Direct cause of death

	Total		1st TKI		1st RT		
Cause of death	*N* = 79	%	*N* = 49	%	*N* = 30	%	*P*‐value
Intracranial lesions	18	23%	10	20%	8	27%	0.5855
Extracranial lesions	28	35%	18	37%	10	33%	0.8123
Both intra‐ and extracranial lesions	13	16%	9	18%	4	13%	0.7565
Treatment related death	2	3%	1	2%	1	3%	1.0000
Other disease	6	8%	4	8%	2	7%	1.0000
Not available	12	15%	7	14%	5	17%	—

RT, radiotherapy; TKI, tyrosine kinase inhibitor.

### Subgroup analysis of OS

In univariate analysis for OS of the entire cohort, ECOG‐PS < 2 (*P* < 0.0001), KPS ≥70 (*P* = 0.017), postoperative recurrence and recurrence after treatment for locally advanced cancer (*P* = 0.005), absence of extracranial metastases (*P* = 0.0001), and GPA ≥2 (*P* = 0.0021) were associated with prolonged OS (Table 5). In multivariate analysis for OS, ECOG‐PS < 2 (*P* = 0.0192) and absence of extracranial metastases (*P* = 0.0044) were independent factors associated with prolonged OS.

**Table 5:** Univariable and multivariable analyses of overall survival

		Univariable		Multivariable	
Variable	Risk group	HR (95%CI)	*P*‐value	HR (95%CI)	*P*‐value
Age	< 60 years old	0.79 (0.45–1.32)	0.379		
	< 70 years old	0.82 (0.52–1.30)	0.390		
Sex	Female	0.67 (0.42–1.08)	0.101		
Smoking status	Never	0.81 (0.51–1.31)	0.387		
ECOG performance status	< 2	0.35 (0.22–0.57)	<0.0001	0.39 (0.19–0.85)	0.0192
KPS	≥ 70	0.43 (0.26–0.72)	0.0017	0.78 (0.37–1.74)	0.526
Stage	Recurrence vs. IV	0.43 (0.21–0.79)	0.0050	0.58 (0.28–1.07)	0.0845
Number of BMs	≤ 4	0.69 (0.44–1.12)	0.132		
Max size of BMs	< 15 mm	0.75 (0.47–1.19)	0.213		
Initial brain symptom	No	0.72 (0.44–1.19)	0.196		
Extracranial metastases	No	0.38 (0.23–0.63)	0.0001	0.48 (0.28–0.80)	0.0044
DS‐GPA	≥ 2	0.47 (0.28–0.77)	0.0021	0.82 (0.46–1.43)	0.480
EGFR mutation	Ex 19 del v L858R	0.87 (0.54–1.39)	0.552		
Primary Leptomeningeal metastases	No vs. Yes	0.60 (0.29–1.44)	0.232		
EGFR‐TKI	GEF vs. ERL	0.75 (0.44–1.32)	0.302		
Brain radiotherapy prior to TKI	1st vs. others	0.86 (0.53–1.36)	0.523		

BM, brain metastasis; CI, confidence interval; DS‐GPA, diagnosis‐specific Graded Prognostic Assessment; ECOG, Eastern Cooperative Oncology Group; HR, hazard ratio; KPS, Karnofsky performance status; LM, leptomeningeal metastases; TKI, tyrosine kinase inhibitor.

## Discussion

We retrospectively evaluated the clinical outcomes and background of 104 *EGFR*‐mutant NSCLC patients with CNS metastases who were administered EGFR‐TKIs as a first‐line drug therapy. Patients who received upfront brain RT showed more favorable TTF and CNS‐PFS than did those who received EGFR‐TKI first without upfront brain RT. Notably, in our study, the 1st RT group showed significantly longer TTF, even though the 1st RT population had larger and more symptomatic BMs than did the 1st TKI population. These findings suggested that this population benefited from brain RT prior to EGFR‐TKI.

In previous reports, the median PFS ranged from 6.6 to 11.4 months, the median CNS‐PFS ranged from 14.5 to 19.7 months, the median OS ranged from 13.6 to 31.7 months, the ORR ranged from 68.2 to 87.8%, and the CNS‐RR ranged from 36.5 to 82.6% for *EGFR*‐mutant patients with BMs who were treated with EGFR‐TKI.^14^, ^15^, ^21^, ^22^, ^23^ In our study, TTF, CNS‐PFS, and OS were similar to those data. In contrast, the ORR of 62% and CNS‐RR of 37% were relatively low. This result can be explained by data unavailability; that is, the treatment response of eight patients in ORR and 13 patients in CNS‐RR were not evaluated, although they were included in the study. In addition, regarding CNS‐RR, we considered irradiated brain lesions as unmeasurable unless there had been demonstrated progression in the lesions according to RECIST, resulting in an evaluation of false SD, even though those lesions were shrinking. In fact, the disease control rate of 81% in this study as well as the rates of TTF, CNS‐PFS, and OS were not inferior to the results from previous reports.

In the current study, patients were treated with first and second generation EGFR‐TKI as a first‐line chemotherapy. Recently, evidence regarding the efficacy of a third generation TKI, osimertinib, which selectively inhibits both EGFR‐TKI‐sensitizing and T790M resistance mutations, has accumulated. In a phase III trial, osimertinib showed significantly longer PFS and less toxicity than did first generation EGFR‐TKIs as a first‐line therapy for *EGFR*‐mutant NSCLC patients.^24^ In addition, compared to first generation EGFR‐TKIs, osimertinib demonstrated significantly longer PFS in patients with BMs.^24^ These favorable antitumor effects in patients with BMs might be due to the high concentration of osimertinib in cerebrospinal fluid.^25^ Nonetheless, further prospective trials are required to evaluate the efficacy of brain RT in *EGFR*‐mutant patients with CNS metastases before the initiation of osimertinib.

Several mechanisms have been proposed to explain the effect of combinational therapy with radiation and EGFR‐TKI. Previous studies have shown the high radiosensitivity of *EGFR*‐mutant NSCLC.^26^, ^27^ Moreover, WBRT or focal RT can cause early and delayed blood brain barrier disruption, which presumably leads to an increase in TKI permeability.^28^ In addition, EGFR‐TKIs could enhance the radiation response at several levels, including cell cycle arrest, apoptosis induction, accelerated cellular repopulation, and DNA damage repair.^29^ Based on these biological rationales, radiation and EGFR‐TKI are expected to provide good combinational therapy. In our study, the 1st RT population showed more favorable TTF and CNS‐PFS than did the 1st TKI population. However, OS showed no significant difference between these groups. In fact, of the 65 1st TKI patients, 43 (66%) received second line drug therapy, while 21 of the 39 1st RT patients (54%) received second line drug therapy (*P* = 0.2207). Of the 43 1st TKI patients who received second line drug therapy, 28 (65%) received platinum doublet therapy as the second line treatment, while 11 of the 21 1st RT patients (52%) received that (*P* = 0.2874). Of the 65 1st TKI patients, 34 (52%) received brain RT at any point during their clinical course after EGFR TKI therapy. Thus, the majority of the patients in both groups received subsequent drug therapy, and the majority of the 1st TKI group received some kind of brain RT eventually. The absence of significant difference in OS might be attributed to these successful subsequent therapies or salvage local therapies. In the meantime, a recent meta‐analysis showed that upfront RT followed by TKI had longer OS as well as CNS‐PFS, especially for patients with limited number of BMs.^30^

Recent real world data collected by EORTC Lung Cancer Group showed that NSCLC patients with driver mutations generally received more intensive local treatments.^31^ Further, a recent large‐scale retrospective study of *EGFR*‐mutant NSCLC patients with BMs reported that survival time was improved by the combination of upfront SRS and EGFR‐TKI compared to that by upfront WBRT and EGFR‐TKI or TKI monotherapy (46 months vs. 30 months vs. 25 months, *P* < 0.001).^20^ Interestingly, this tendency was observed in patients with a favorable prognosis, DS‐GPA 2–4, and in those with a less favorable prognosis, DS‐GPA 0–1.5, suggesting that the improved OS seen in the upfront SRS group was not secondary to selection bias or differences between patient cohorts. In our study, there was no significant difference in TTF, CNS‐PFS and OS between patients who received STI and those who received WBRT as brain RT prior to EGFR‐TKI (Fig S2). In any case, a prospective study comparing 1st STI followed by EGFR‐TKI with 1st TKI in *EGFR*‐mutant NSCLC patients with BMs is required. Moreover, whether WBRT should be added to STI as a treatment option in patients with BMs remains unclear.

Brain radiotherapy could deteriorate QOL and impair cognitive function in patients. In our retrospective study, we could not analyze toxicities of brain radiotherapy due to lack of data. According to a systematic review reported by Hendriks *et al*. most of nine trials showed no increased neurotoxicity in TKI plus brain radiotherapy compared to TKI alone.^32^ Whereas, another retrospective study reported increase of memory impairment in TKI plus WBRT compared to TKI alone.^17^ Two randomized phase 3 studies of solid tumors with one to three BMs showed that SRS plus WBRT impaired health‐related QOL and cognitive function, respectively, and did not improve OS compared to SRS alone.^33^, ^34^ In this regard, WBRT in addition to SRS may not be routinely recommended in patients with limited number of BMs.

Sperduto *et al*. developed DS‐GPA to evaluate the prognosis of patients with BMs^35^; DS‐GPA is now used as a prognostic factor in an American Society for Radiation Oncology guideline for radiotherapeutic and surgical management for newly diagnosed BMs.^36^ For NSCLC, DS‐GPA consists of four prognostic factors, including age, KPS, extracranial metastases, and number of BMs. In our study, univariate and multivariate analyses indicated that the existence of extracranial metastases and poor PS (ECOG‐PS ≥2) are independent poor prognostic factors for OS in *EGFR*‐mutant NSCLCs. These findings almost corresponded to DS‐GPA as they were related to the factors comprising DS‐GPA. Two recent phase II studies have shown a survival benefit from WBRT on SRS in patients with good DS‐PGA,^37^, ^38^ suggesting that potential long‐term survivors could receive the benefit of WBRT. Similar to these findings, our patients younger than 70 or with good PS in the 1st RT group had longer TTF than those in the 1st TKI group (Fig S1). However, OS in the 1st RT group was not significantly prolonged. Whether the addition of WBRT to STI is especially beneficial for potential long‐time survivors, for example, those with good DS‐GPA, should be determined. Recently, Sperduto *et al*. updated GPA for NSCLC by adding the factor of gene status to DS‐GPA^39^; this change apparently contributes to an improved reflection of actual clinical practice.

Our study has several limitations that should be acknowledged. First, owing to its retrospective nature, which lacks randomization, bias might yield a difference in clinical outcomes between the groups. Second, the choice of treatment was not random because it was determined by both physicians and patients in as many as 10 different facilities whose location or equipment varies. Third, the sample size of this study was small, which may have affected its statistical power. Fourth, adverse events were not analyzed due to the lack of clinical data. In particular, a complication of leukoencephalopathy was supposed to be monitored because it strongly triggers neurocognitive dysfunction and a decrease in QOL. Leukoencephalopathy can be an important factor to consider when determining treatment.

In conclusion, our retrospective analysis demonstrated a favorable response of CNS metastases treated with EGFR‐TKI in *EGFR*‐mutant NSCLC patients. Furthermore, prolonged TTF and CNS‐PFS were observed with upfront brain RT. Further research through prospective studies is needed to determine the optimal timing of brain RT for the most appropriate population in *EGFR*‐mutant NSCLC patients with CNS metastases.

## Disclosure

Satoshi Watanabe received honoraria from AstraZeneca, Chugai Pharma, Bristol‐Myers, Boehringer Ingelheim, Ono Pharmaceutical, Taiho Pharmaceutical; Tetsuya Abe received honoraria from AstraZeneca, Chugai Pharma, Boehringer Ingelheim, Ono Pharmaceutical, Eli Lilly Japan, Novartis, Taiho Pharmaceutical; Kosuke Ichikawa received honoraria from AstraZeneca, Chugai Pharma, Bristol‐Myers, Boehringer Ingelheim, Ono Pharmaceutical, Taiho Pharmaceutical; Kenichi Koyama received honoraria from AstraZeneca, MSD, Chugai Pharma, Ono Pharmaceutical, Eli Lilly, Iyaku Jounal; Satoru Miura received honoraria from Bristol‐Myer, Ono Pharmaceutical, Boehringer Ingelheim, Eli Lilly, MSD, Chugai Pharma, AstraZeneca, Taiho Pharmaceutical, Kyowa Hakko Kirin, Mochida Pharmaceutical; Hiroshi Tanaka received honoraria from AstraZeneca, Chugai Pharma, Boehringer Ingelheim, Ono Pharmaceutical, Bristol‐Myers, Taiho Pharmaceutical, Eli Lilly, MSD, Astellas Pharmaceutical, Pfizer, Takeda pharmaceutical, Novartis, Merck; Masaaki Okajima received honoraria from AstraZeneca, Chugai Pharma, Bristol‐Myers, Boehringer Ingelheim, Ono Pharmaceutical, Taiho Pharmaceutical, MSD; Hiroki Tsukada received honoraria from AstraZeneca, Taisho Toyama Pharmaceutical, Astellas Pharma, Boehringer Ingelheim, Ono Pharmaceutical, Meiji Seika Pharma, Daiichi Sankyo, Shionogi, GlaxoSmithKline, Novartis, MSD, Sumitomo Dainippon Pharma, Takeda Pharmaceutical, Kyorin Pharmaceutical, Asahi Kasei Pharma, Fujifilm Pharma; Kazuhiro Sato received honoraria from AstraZeneca, Boehringer Ingelheim, Ono Pharmaceutical, MSD, Pfizer; Toshiaki Kikuchi received honoraria from Chugai Pharma, Boehringer Ingelheim, Eli Lilly Japan. The other authors indicated no financial relationships.

## Supporting information


**Figure S1** Forest plot of hazard ratios (HR) for time to treatment failure (TTF) by baseline characteristics in *EGFR*‐mutant NSCLC patients with brain metastases who received EGFR tyrosine kinase inhibitors with upfront radiotherapy (RT) and EGFR tyrosine kinase inhibitors without upfront RT as first‐line therapy. ECOG‐PS, Eastern Cooperative Oncology Group performance status; KPS, Karnofsky performance status; BM, brain metastasis; DS‐GPA, diagnosis‐specific Graded Prognostic Assessment; TKI, tyrosine kinase inhibitor; RT, radiotherapy; HR, hazard ratio; CI, confidence interval.

**Figure S2** Kaplan‐Meier analysis of time to treatment failure, CNS‐PFS, and overall survival comparing in the patients treated with upfront brain STI, in those treated with upfront WBRT and in those treated without upfront radiotherapy (A, B, and C). CNS, central nervous system; PFS, progression‐free survival; STI, stereotactic irradiation; WBRT, whole brain radiotherapy; TKI, tyrosine kinase inhibitor.
