# Supplementary figures and images for: Efficacy of EGFR‐TKIs with or without upfront brain radiotherapy for EGFR‐mutant NSCLC patients with central nervous system metastases
Source: Thorac Cancer. 2019 Sep 10;10(11):2106–16. doi: 10.1111/1759-7714.13189 (PMC6825912; doi:10.1111/1759-7714.13189)

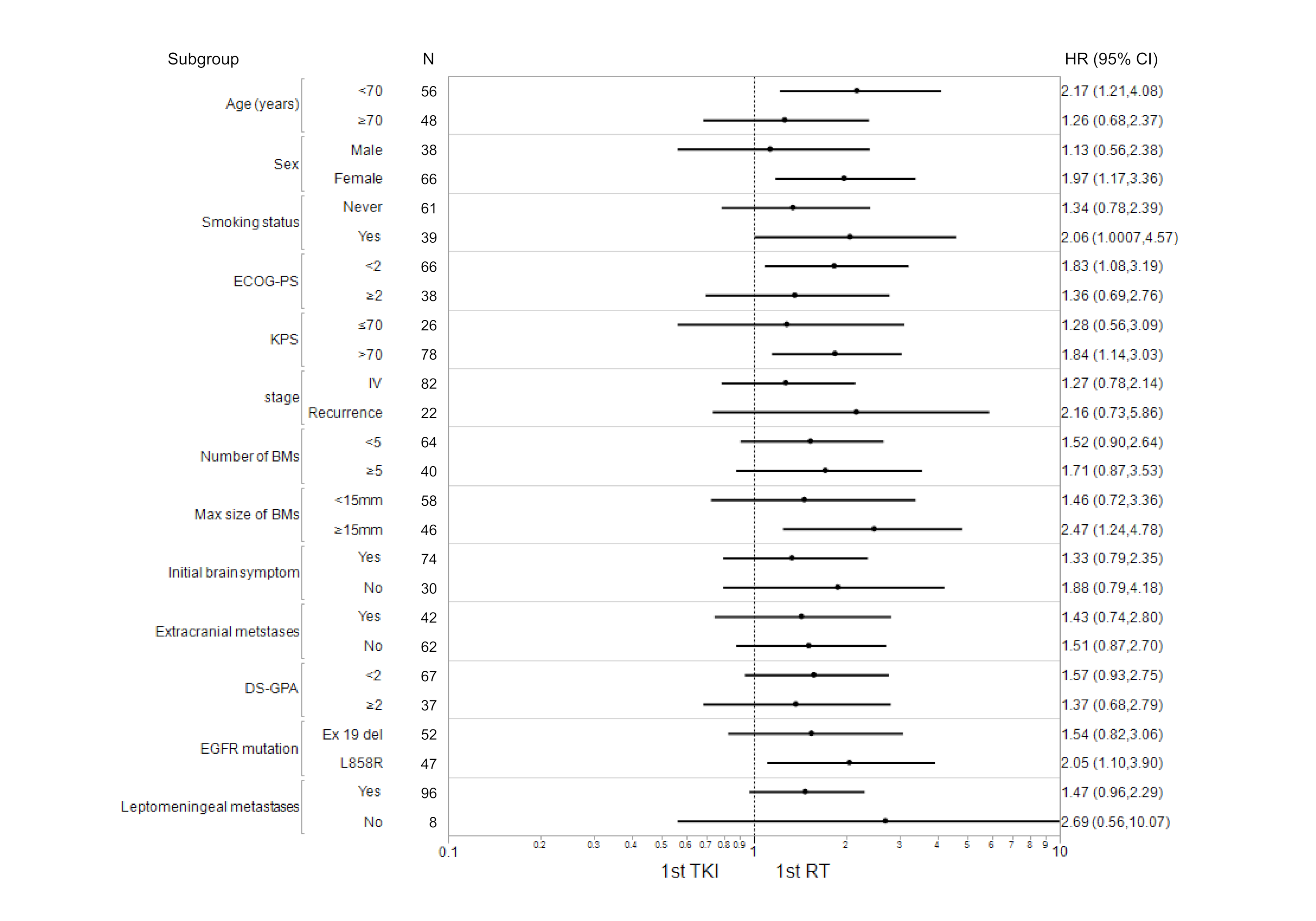

Supplement: Supplementary file 1 — Figure S1 Forest plot of hazard ratios (HR) for time to treatment failure (TTF) by baseline characteristics in EGFR‐mutant NSCLC patients with brain metastases who received EGFR tyrosine kinase inhibitors with upfront radiotherapy (RT) and EGFR tyrosine kinase inhibitors without upfront RT as first‐line therapy. ECOG‐PS, Eastern Cooperative Oncology Group performance status; KPS, Karnofsky performance status; BM, brain metastasis; DS‐GPA, diagnosis‐specific Graded Prognostic Assessment; TKI, tyrosine kinase inhibitor; RT, radiotherapy; HR, hazard ratio; CI, confidence interval. [file TCA-10-2106-s001.tif]

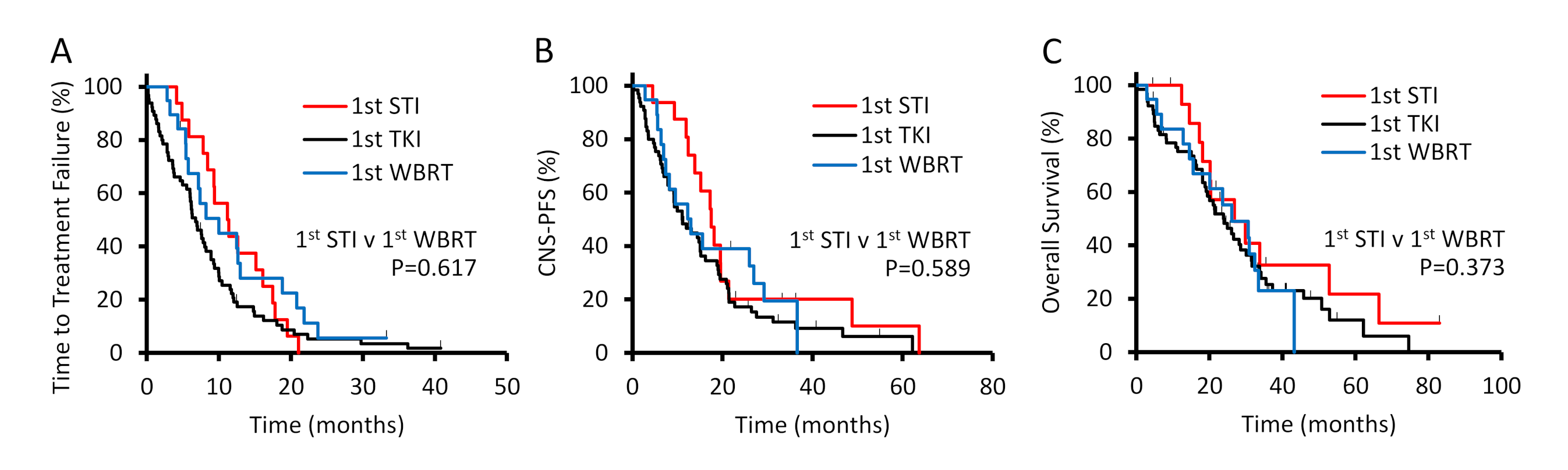

Supplement: Supplementary file 2 — Figure S2 Kaplan‐Meier analysis of time to treatment failure, CNS‐PFS, and overall survival comparing in the patients treated with upfront brain STI, in those treated with upfront WBRT and in those treated without upfront radiotherapy (A, B, and C). CNS, central nervous system; PFS, progression‐free survival; STI, stereotactic irradiation; WBRT, whole brain radiotherapy; TKI, tyrosine kinase inhibitor. [file TCA-10-2106-s002.tif]
